# Proteomic Analysis of *Apis cerana* and *Apis mellifera* Larvae Fed with Heterospecific Royal Jelly and by CSBV Challenge

**DOI:** 10.1371/journal.pone.0102663

**Published:** 2014-08-07

**Authors:** Yi Zhang, Guozhi Zhang, Xiu Huang, Richou Han

**Affiliations:** Guangdong Entomological Institute, Guangzhou, China; National Central University, Taiwan

## Abstract

Chinese honeybee *Apis cerana* (*Ac*) is one of the major Asian honeybee species for local apiculture. However, *Ac* is frequently damaged by Chinese sacbrood virus (CSBV), whereas *Apis mellifera* (*Am*) is usually resistant to it. Heterospecific royal jelly (RJ) breeding in two honeybee species may result in morphological and genetic modification. Nevertheless, knowledge on the resistant mechanism of *Am* to this deadly disease is still unknown. In the present study, heterospecific RJ breeding was conducted to determine the effects of food change on the larval mortality after CSBV infection at early larval stage. 2-DE and MALDI-TOF/TOF MS proteomic technology was employed to unravel the molecular event of the bees under heterospecific RJ breeding and CSBV challenge. The change of *Ac* larval food from RJC to RJM could enhance the bee resistance to CSBV. The mortality rate of *Ac* larvae after CSBV infection was much higher when the larvae were fed with RJC compared with the larvae fed with RJM. There were 101 proteins with altered expressions after heterospecific RJ breeding and viral infection. In *Ac* larvae, 6 differential expression proteins were identified from heterospecific RJ breeding only, 21 differential expression proteins from CSBV challenge only and 7 differential expression proteins from heterospecific RJ breeding plus CSBV challenge. In *Am* larvae, 17 differential expression proteins were identified from heterospecific RJ breeding only, 26 differential expression proteins from CSBV challenge only and 24 differential expression proteins from heterospecific RJ breeding plus CSBV challenge. The RJM may protect *Ac* larvae from CSBV infection, probably by activating the genes in energy metabolism pathways, antioxidation and ubiquitin-proteasome system. The present results, for the first time, comprehensively descript the molecular events of the viral infection of *Ac* and *Am* after heterospecific RJ breeding and are potentially useful for establishing CSBV resistant populations of *Ac* for apiculture.

## Introduction

*Apis mellifera* (*Am*) and *A. cerana* (*Ac*) are major honey bee species in the global beekeeping industry [1], [2]. They are heavily infected by different vital viruses [3], [4]. Chinese sacbrood virus (CSBV) is the most stricken pathogen of *Ac*, which results in severe and deadly infections of the colony and eventually losses of the entire colony [5]. As a small RNA virus (picorna-like virus) that has an icosahedral virion with a diameter of 26–30 nm, CSBV genome consists of a single positive-strand RNA molecule with 8.8 kilo bases (kb) [7]. Although CSBV shows a close genetic relationship to its western counterpart sacbrood virus (SBV), there is no cross infection [8]. Since this viral disease broke out in 1972 in southern China [8], [9], some efforts have been made to study this virus, such as diagnostic methods (electron microscopy, enzyme-linked immunosorbent assay and reverse transcription-polymerase chain reaction (RT-PCR)) [10], and the control of this disease by RNA interference [11]. It is interesting that *A. mellifera* is not sensitive to CSBV in general beekeeping practice [12]. Similar to all other insects, the honeybees lack a classically adaptive immune system as in the case of mammalian [6]. To survive, they have evolved cellular and humoral immune responses to cope with microbial infections. *A. mellifera* may develop cellular and humoral immune responses to various pathogens such as bacteria [13], [14], viruses [15], microsporidian [16]–[18] and *Varroa* mites [19], [20]. Usually, the viral infection causes cell apoptosis, tissue damage and even functional disorder of the whole organism and all these changes can be further reflected in proteome alteration [21]. Recent study revealed the pathological mechanism of Chinese sacbrood disease to *Ac* [6]. However, no information on the difference in the immune responses of two bee species to CSBV is reported.

In the honey bees, queen and workers have different behavior and reproductive capacity despite possessing the same genome. The primary substance that leads to this differentiation is royal jelly (RJ), which contains a range of proteins, carbohydrates, lipids, minerals, vitamins, and a large number of bioactive substances, especially immunological peptides and antibacterial proteins [22]–[24]. Major Royal Jelly Proteins (MRJPs) are the prime RJ ingredients, which are crucial in regulating reproductive maturation [22]. There are quantitative differences in nucleic acids and protein composition in fresh RJ between *Am* and *Ac* [24]. The most recent discovery is that RJ contains microRNAs which may play a role in caste differentiation [25]–[27]. Whether the RJ also influences the resistant behavior of *Am* to CSBV is unknown. The molecular event for CSBV resistance of *Am* needs to be also determined.

Two-dimensional gel electrophoresis (2-DE) based proteomics technology and matrix assisted laser desorption ionization time-of-flight mass spectrometry (MALDI-TOF MS) are powerful molecular tools. In recent years, proteomic techniques have been successfully used to profile the proteome change in insect growth and development [6], [28]–[30]. The present study employed gel-based (2-DE) and shotgun proteomic (label-free LC-MS based) strategies, which have complementary natures, to gain an in-depth understanding of the resistant mechanism of *Am* to the fatal CSBV disease by comparison of the proteome-wide change of the healthy and sick worker larvae of both *Am* and *Ac* fed with RJ either from *Am* (RJM) or *Ac* (RJC).

In the present study, we performed comparison analyses of the proteome in *Ac* and *Am* after heterospecific RJ breeding (i.e. *Am* larvae fed with RJC or *Ac* larvae fed with RJM), and then CSBV challenge, by two-dimensional electrophoresis and MALDI-TOF MS analysis, to systematically search for the molecular basis of the crossbreeding and CSBV challenge. A total of 101 differentially expressed nonredundant proteins (≥3 folds change) were identified. Furthermore, gene expression patterns in two bee species were investigated at mRNA levels. The results show that differential expression proteins are involved in the regulation of metabolism and response to CSBV challenge.

## Materials and Methods

### Ethics Statement

Honey bee colonies (*Am* and *Ac*) were raised at Conghua, Guangdong Province, China (113°17' E, 23°8'' N), by Guangdong Entomological Institute, by standard beekeeping techniques. According to the present regulation from Ministry of Agriculture *Am* and *Ac* used in this studies are not endangered or protected species. No specific permissions were required for performing these experiments.

### Honey Bees

To obtain age controlled second instar larvae, the queen was caged on a comb and left to lay eggs for 6 h. Twenty hours after larval eclosion, or 92 h after oviposition, the comb containing second instar larvae was retrieved from the colony, and placed in the laboratory for treatments at 32–34°C. All the larvae used in this study were detected by Reverse Transcription-Polymerase Chain Reaction (RT-PCR) method for the absence of the following viruses, black queen cell virus (BQCV), chronic bee paralysis virus (CBPV), deformed wing virus (DWV), kashmir bee virus (KBV), Chinese sacbrood virus (CSBV) and Israeli acute paralysis virus (IAPV), with the primers from Ai *et al*. [4]. RNA was extracted using Trizol Reagent (Invitrogen, Carlsbad, USA) and cDNA synthesis was performed using PrimeScript 1st Strand cDNA Synthesis Kit (TaKaRa, Kyoto, Japan). Amplification profile of PCR consisted of an initial 2-min denaturation at 94°C, followed by 35 cycles of 30 s at 95°C, 30 s at 55°C, 1 min at 72°C and finally 7-min cycle at 72°C. No signs of clinical American foulbrood or other viral disease were observed in these larvae.

### Virus

CSBV-infected larvae with typical symptom were collected from an apiary at Xinhua Village from Hunan Province, and kept at - 80°C for less than 10 days before use. The presence of CSBV virus was confirmed by observation of the morphological symptom under electronic microscopy and RT-PCR according to Chen *et al*. and Yan *et al*. [3], [10].

To obtain CSBV, the larvae (approximately 0.2 g) infected by CSBV were ground in 6 mL sterile phosphate-buffered solution (PBS) (137 mM NaCl, 2.7 mM KCl, 10 mM Na_2_HPO_4_, 1.8 mM KH_2_PO_4_, pH 7.4) with a sterile grinder. The resulting solution was centrifuged at 14 000 g at 4°C for 10 min, and the supernatant was further passed through a 0.45 µm cell filter first, then through a 0.22 µm cell filter [31]. The CSBV concentration was quantitated by absolute quantification assay [11]. CSBV polyprotein gene (SBV1) was amplified to detect copy number of CSBV according to Liu *et al*. [11]. PCR reactions were carried out in triplicate in an Mx3000P Real-Time PCR System (Stratagene, California, USA), using SYBR_Green (Brilliant II SYBR-Green QPCR Master Mix; Stratagene, California, USA). The CSBV concentration for the experiments was approximately 7.9×10^5^ copies/mL.

### Harvest of RJM and RJC

RJM and RJC were produced according to standard practices in China [32]. Briefly, the queen was confined inside a queen excluding cage. Queen cups with young larvae (one-day old) were introduced into the colony and allowed to be fed by workers for 2 days. 3-day old larvae were first carefully removed by using either a grafting tool or a pair of forceps, then the royal jelly was removed by using a spatula.

### Larvae Fed with RJM or RJC and Challenged with CSBV

To determine the effects of the changing food (RJs) on the mortality and protein expression of the third instar larvae, which are the most sensitive stage to CSBV infection, the second instar larvae of *Ac* and *Am* were collected carefully from the combs, respectively transferred to 96-cell culture plates containing 30 µL RJC or RJM, in each cell, then placed in a cabinet (Sanyo, Tokyo, Japan) at 80% relative humidity at 32–34°C. After 24 h, the third instar larvae of both bee species fed with RJM or RJC were transferred to new plates, and challenged with 30 µL CSBV solution per cell, prepared as described above. After 8 h, the larvae were washed with sterile distilled water for 3 times, and kept at −80°C for use. 8 treatments (Table 1) were established with 3 replicates containing 20 larvae in each replicate. Each 20 larvae were pooled as one biological replicate and three independent biological replicates were produced.

**Table 1:** Treatments of the two honey bee species by RJs and CSBV.

No.	Honey bees	Treats	Abbreviation
**A**	*A. cerana*	fed on *A. cerana* royal jelly	AC-RJC
**B**		fed on *A. cerana* royal jelly, and challenged with CSBV	AC-RJC+CSBV
**C**		fed on *A. mellifera* royal jelly	AC-RJM
**D**		fed on *A. mellifera* royal jelly, and challenged with CSBV	AC-RJM+CSBV
**E**	*A. mellifera*	fed on *A. cerana* royal jelly	AM-RJC
**F**		fed on *A. cerana* royal jelly, and challenged with CSBV	AM-RJC+CSBV
**G**		fed on *A. mellifera* royal jelly	AM-RJM
**H**		fed on *A. mellifera* royal jelly, and challenged with CSBV	AM-RJM+CSBV

The same treatments were also established to examine the mortality of bee larvae fed with RJM or RJC and challenged by CSBV after 24 h. The death of the larvae was confirmed when they had no response after stimulating with a soft tip, and by the color change of their bodies (from white to yellow or even dark).

### Protein Extraction and 2-DE

Larval protein extractions were carried out according to the previously described method with some modifications [33], [34].

Bee larvae were manually homogenized for 30 min and sonicated for 2 min on ice in lysis buffer (8 M urea, 0.2% w/v Bio-Lyte 3/10 Ampholyte (Bio-Rad, USA), 4% CHAPS, 65 mM DTT) containing Protease Inhibitor Cocktail (Calbiochem, Germany)(w/l = 1 mg/4 µL). The mixture was centrifuged 15 000 g at 4°C for 30 min, and the supernatant was collected. The resulting precipitates were treated three times with the same procedures. All the supernatants were centrifuged 15 000 g at 4°C for 60 min. The final supernatant was used or stored at −80°C for 2-DE. Protein concentrations were determined by the Bradford method using Modified Bradford Protein Assay Kit (Sangon, Shanghai, China).

The 2-DE was performed according to the methods described previouslyand the manufacturer's instruction [35]. The first dimension (isoelectric focusing) was conducted using the IPGphor IEF system (Bio-Rad, California, USA) at 20°C. For analytical gels, 350 µg protein was solubilized in 400 µL rehydration solution (8 M urea, 0.2% w/v Bio-Lyte 3/10 Ampholyte, 4% CHAPS, 65 mM DTT, 0.001% w/v bromophenol blue), and loaded onto a 17 cm pH 3–10 NL IPG strip (Bio-Rad). Focusing was performed for 13 h at 50 V, 1 h at 500 V, 1 h at 1000 V, and 30 min and 6 h at 8000 V (total = 48 kVh). The IPG strips were equilibrated as previously described. The second dimension was performed with 13% (w/v) SDS-polyacrylamide gels using the Protean II xi 2D Multicell system (Bio-Rad, California, USA). Proteins were stained with silver nitrate, and gels were digitized using Image ScannerII (Amersham Biosciences, USA). Digitized 2-DE gel patterns were edited and matched using the PDQUEST V8.0.1 software package (PDI, Humington Station). Triplicate experiments were run to confirm the reproducibility of results.

### Protein Identification by MALDI-TOF/TOF MS

Protein digestion and peptide extraction were done according to our previously established protocol [36]. Spots of interest in gels staining with silver nitrate were cut out, washed, reduced, S-alkylated with iodoacetamide and in-gel digested at 37°C overnight with sequencing grade porcine trypsin (Promega, USA). After extraction in extractant of 50% ACN (Fisher, Waltham, MA,USA) and 2.5% TFA (Sigma, St Louis, MO, USA), peptide mixtures were analyzed using a saturated solution of 5 mg/mL α-cyano-4-hydroxycinnamic acid (CHCA, Sigma, St Louis, MO, USA) in ACN containing 0.1% TFA (Sigma, St Louis, MO, USA) (50/50 v/v) using a 4800 Proteomics Analyzer equipped with matrix assisted laser desorption ionization time-of-flight mass spectrometry (MALDI-TOF MS) (Applied Biosystems, Framingham, MA, USA). For MS calibration, the trypsin autolysis peptides were used as internal calibrants. Monoisotopic peak masses were automatically determined within the mass range of 800–4000 Da, with a minimum S/N of 50. Five of the most intense ion signals were selected as precursors for MS/MS acquisition. Combined MS and MS/MS queries were performed with the MASCOT search engine (V2.1, Matrix Science, UK) embedded in GPS-Explorer Software (V3.6, Applied Biosystems, Framingham, MA, USA), using the *A. mellifera* database (Gene DB). MASCOT protein scores (based on combined MS and MS/MS spectra) of greater than 61 were considered statistically significant (*P*≤0.05). The individual MS/MS spectrum with statistically significant (confidence interval >95%) best ion score (based on MS/MS spectra) were also accepted.

Based on the UniProt Knowledgebase (http://www.uniprot. org/), identified proteins were submitted to KEGG (Kyoto Encyclopedia of Genes and Genomes) and classified according to KEGG pathway maps (http://www.genome.jp/kegg/pathway.html) and Gene Ontology (http://www.geneontology.org/), grouped on the basis of their biological process of GO terms (http://www.blast2go.com) [19]. Protein interaction networks of the differentially regulated proteins were analyzed using the online database resource Search Tool for the Retrieval of Interacting Genes (STRING: http://string-db.org) [37], [38].

### Quantitative Real-Time PCR (qRT-PCR)

To verify the gene expression of differentially expressed proteins, 11 proteins (FBA, LOC408516, MDH, SOD, Jafrac1, LOC725646, AGO1, Che-3, Ald, Pxd and Tpi) were randomly selected for qRT-PCR analysis of their encoding genes by quantitative real time PCR, according to our previously method with some modifications [19]. Briefly, total RNA was isolated using Trizol reagent (Invitrogen, Carlsbad, USA). The RNA was dissolved in diethylpyrocarbonate (DEPC) treated water and quantified by measuring ultraviolet absorbance at 260 and 280 nm. Reverse transcription was performed on 1 µg of total RNA using PrimeScript RT reagent Kit with gDNA Eraser (Takara, Kyoto, Japan). The gene-specific primers for the analysis are listed in Table 2. Expression of *actin* gene (GI: 406122) was used as an internal control. The efficiency of each primer set was first validated by constructing a standard curve through five serial dilutions. PCR reactions were carried out in triplicate in an Mx3000P Real-Time PCR System (Stratagene, California, USA), using SYBRGreen (SYBR Premix Ex Taq II, Takara, Kyoto, Japan). A control without template was included in all batches. The PCR program began with a single cycle at 95°C for 10 min, 40 cycles at 95°C for 15 s and 48°C for 60 s. Afterwards, the PCR products were heated to 95°C for 15 s, cooled to 48°C for 15 s and heated to 95°C for 15 s, in order to measure the dissociation curves and to determine a unique PCR product for each gene. mRNA levels were calculated relative to *actin* expression using the Mx3000P Software (version 4.1) (Agilent, California, USA). The fold change was calculated using the 2^−ΔΔCt^ method. Each sample was analyzed independently and processed in triplicate.

**Table 2:** Primers for qRT-CR analysis of 12 differentially expressed genes.

Product name	Gene ID	Forward primer sequence	Reverse primer sequence	Amplicon size (bp)
Actin	gi|406122	ATGCCAACACTGTCCTTTCTGG	GACCCACCAATCCATACGGA	150
FBA	gi|66526635	TTTCTAGTATCTAAAGCATG	ACTGCTATTGCTACTGCT	228
LOC408516	gi|66524882	GATCCAGATGCACCAAG	GGACAGAATCGTGGAAA	235
MDH	gi|66513092	AAGGCTGGCACAGGTTC	TAAATGCAGCGATCCCA	239
SOD	gi|33089104	ACTTGTCGTTCCGTGTA	CACATTTCAACCCATTA	229
Jafrac1	gi|66548188	AAACTCATTGCAGCATC	GGGAGGTCTGTTGATGA	250
LOC725646	gi|110755974	ACCCGAATTGTACTTTA	TAGATGAAGGTGCTCAA	221
AGO1	gi|110777044	TGGCCCAGATCAAGTAGAGC	AATTTGATAGCGTTTGTGGTGAT	200
Che-3	gi|110757336	TCCTGTCCGAATTTTTACCTGT	GAAGCTGCGTTTGCGTCTA	250
Ald	gi|110748949	TGCGTACTGTTCCACCT	GAGGGCTAAGGCTAACA	233
Pxd	gi|110757934	CGCTGTTCCAAGAGGCT	ACGACAAACGCCAGATC	250
Tpi	gi|148224276	TCAGTAACAGCAGGAAAT	TTTATGTCATGTTCCTGCAA	269

### Western Blot

To further verify the variation tendency of differentially expressed proteins identified by the proteomic approaches, glyceradehyde-3-phosphate dehydrogenase 2 isofome 1(GAPDH), fmarylacetoacetase hydrolase (FAH), heat shock protein (Hsp)60, phosphoglycerate kinase (PGK), triosephosphate isomerase 1 (Tpi), CuZn superoxide dismutase (SOD) and fructose-bisphophate aldolase (FBA) were randomly selected for Western Blot analysis by the method of Han *et al*. [6] with minor modifications. Briefly, equal amount of protein sample (30 µg/lane) were separated by stacking (5%) and separating (12%) SDS-PAGE (sodium dodecyl sulfate polyacrylamide gel electrophoresis) gels and then transferred to a nitrocellulose transfer membrane (0.2 µm pore size) (Invitrogen, Eugene, OR) using the iBlot apparatus (Invitrogen, Eugene, OR). After blocking, the membranes were incubated over night at 4°C with primary rabbit polyclonal antibodies of anti-GAPDH, FAH, Hsp60, PGK, Tpi, SOD, and FBA antibodies (Abcam, Cambridge, MA) at a dilution of 1∶1000. Following three washes, the membranes were further incubated with horseradish peroxidase-conjugated rabbit anti-goat secondary antibody (BOSTER, Wuhan, China) at a dilution of 1∶2000 for 1 h. Immunoreactive protein bands were detected using the DAB Western Blotting Substrate (BOSTER, Wuhan, China) and quantified by densitometry using Quantity-one image analysis system (Bio-Rad, Hercules, CA). Actin was detected simultaneously as loading control of the analysis, which immunoreactived by BCIP/NBT Western Blotting Substrate (BOSTER, Wuhan, China). Quantification of the protein bands was conducted by scanning the films and importing the images into the Quantity One 1-D analysis software (Bio-Rad, Hercules, CA). Scanning densitometry was used for semiquantitative analysis of the data.

### Data Analysis

The statistical analysis of gene expression was performed by one-way ANOVA test followed by Tukey's test for pair comparisons (SPSS version 16.0, SPSS, Inc.). Comparison of larval mortality differences between different groups was performed by independent samples t test. An error probability *p*<0.05 was considered statistically significant.

## Results

### Effects of RJM and RJC on the Mortality of Two Bee Species after CSBV Challenge

**Figure 1:**
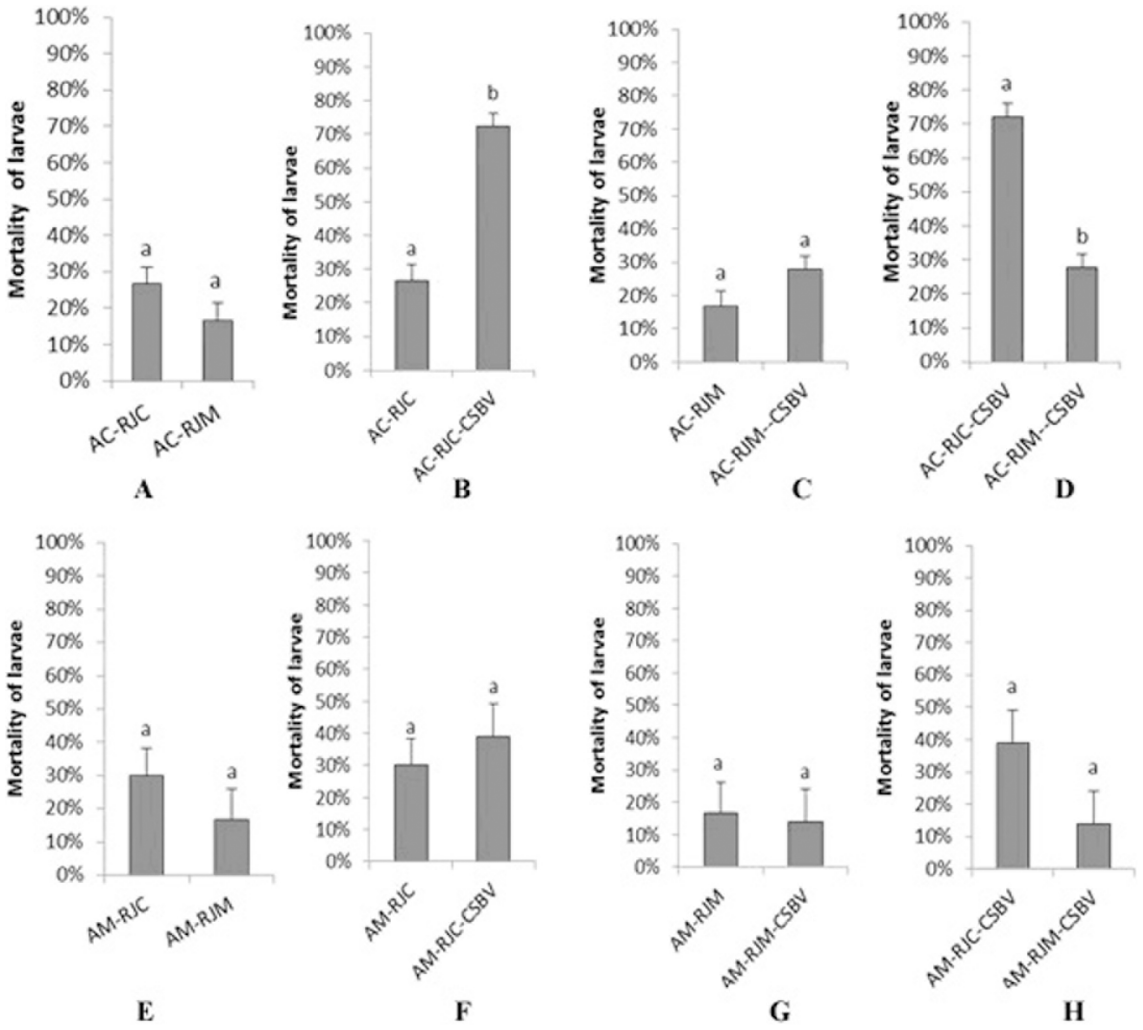
The larval mortalities of *A. cerana* and *A. mellifera* under different treatments after 56 hours. RJC: royal jelly from *A. cerana*; RJM: royal jelly from *A*. mellifera; AC-RJC: 3-day *Ac* larvae fed with RJC. AC-RJM: 3-day *Ac* larvae fed with RJM. AC-RJC+CSBV: 3-day *Ac* larvae fed with RJC, and infected with CSBV. AC-RJM+CSBV: 3-day *Ac* larvae fed with RJM, and infected with CSBV. AM-RJC: 3-day *Am* larvae fed with RJC. AM-RJM: 3-day *Am* larvae fed with RJM. AM-RJC+CSBV: 3-day *Am* larvae fed with RJC, and infected with CSBV. AM-RJM+CSBV: 3-day *Am* larvae fed with RJM, and infected with CSBV. The data are averages from three replicates. The error bars indicate standard deviations. Bars with different letters mean significantly different (P<0.05, independent samples t test).

Experiments were carried out to examine whether RJM would enhance the resistant ability of *Ac* larvae to CSBV infection. The larval mortalities of the treatments after 56 h were indicated in Figure 1. The dead larvae showed the symptom of CSBV infection. Without CSBV infection, the mortalities of *Ac* larvae fed with RJC or RJM were not significantly different (t = 2.040, df = 4, *p* = 0.111; Figure 1 A); meanwhile, the mortalities of *Am* larvae fed with RJM or RJC were also not significantly different (t = 1.572, df = 4, *p* = 0.191; Figure 1 E). This indicated that the RJs did not significantly influence the mortalities of the larvae of two bee species, at least at key early larval stage.

However, the mortality (72.2%) of *Ac* larvae fed with RJC and subsequent CSBV infection was significantly higher than that of the larvae fed with RJC (26.7%) but without CSBV (t = −9.642, df = 3.801, *p* = 0.001; Figure 1 B); When *Ac* larvae were fed with RJM and subsequently challenged by CSBV, their mortality was not significantly different than that of those larvae fed with RJM and without CSBV (t = −2.447, df = 4, *p* = 0.071; Figure 1 C); Interestingly, when *Ac* larvae were fed with RJC or RJM and subsequently challenged with CSBV, the mortality (72.2%) of *Ac* larvae fed with RJC was significantly higher than that of those larvae fed with RJM (27.8%) (t = 10.674, df = 4, *p* = 0.000; Figure 1 D). This indicated that RJM conferred antiviral activity for *Ac* larvae.

When *Am* larvae were fed with RJC or RJM, and subsequently challenged with CSBV, no significant differences in mortalities of *Am* larvae were observed (t = 1.572, df = 4, *p* = 0.191, Figure 1 E; t = −0.941, df = 4, *p* = 0.400, Figure 1 F; t = −0.512, df = 4, *p* = 0.636, Figure 1 G; t = 2.000, df = 4, *p* = 0.116, Figure 1 H). So the RJs did not influence the mortality of *Am* larvae after CSBV challenge. As usual, *Am* is not sensitive to CSBV infection.

### Qualitative Comparisons and Identification of Differentially Expressed Proteins

To determine the effects of the RJ on the protein expression of two bee species after CSBV infection, the protein spots were visualized by 2-DE from the bee larvae of *Ac* and *Am* fed with RJM or RJC, and with RJM or RJC for 24 h and then challenged by CSBV for 8 h. 8 h after CSBV challenged, there was no dead larvae, although the virus was detected in the challenged larvae.

The total proteins were separated on 2-DE gels spanning pH 3–10, silver stained, and analyzed by MS. Protein levels were expressed as percentage volume, which corresponds to the percentage ratio between the volume of a single spot and the total volume of all spots present in a gel. The mean values of spot intensity were calculated using at least three gels. Spots showing more than 15% variation were not considered (Student's test, with 7 degrees of freedom, *P*<0.05). Little deviation was observed in the patterns on replica gels.

**Figure 2:**
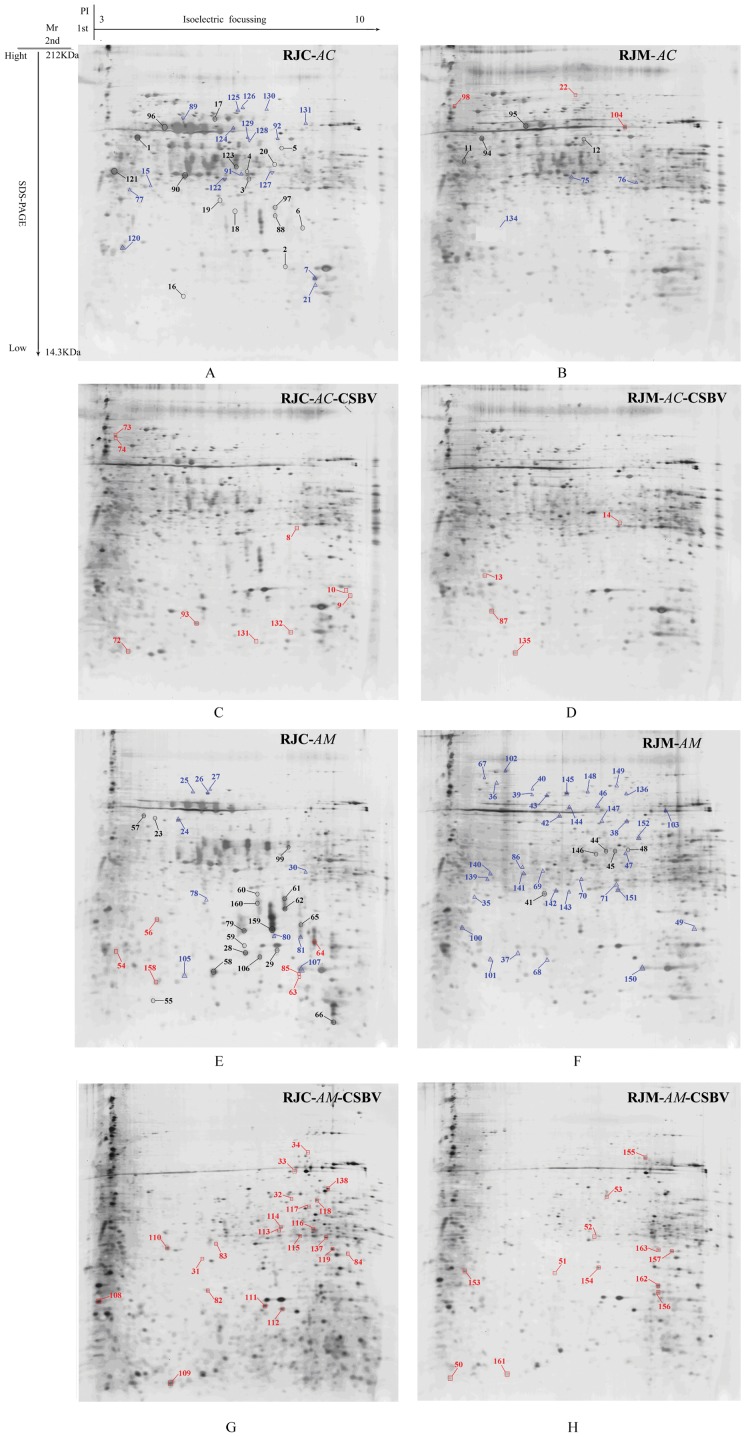
2-DE pictures of *A. cerana* and *A. mellifera* 3-day old larvae fed with RJs and by CSBV infection. RJC: royal jelly from *A. cerana*; RJM: royal jelly from *A. mellifera*; A: 3-day old *Ac* larvae fed with RJC. B: 3-day old *Ac* larvae fed with RJM. C: 3-day old *Ac* larvae fed with RJC and by CSBV infection. D: 3-day old *Ac* larvae fed with RJM and CSBV infection. E: 3-day old *Am* larvae fed with RJC. F: 3-day old *Am* larvae fed with RJM. G: 3-day old *Am* larvae fed with RJC and CSBV infection. H: 3-day old *Am* larvae fed with RJM and by CSBV infection.

530–670 spots were revealed on the silver-stained 2-DE patterns of the total proteins from different larvae, depending on bee species and the treatments (Figure 2). Protein spots were distributed over the 3–10 pH range.

Quantitatively, 296 total spots showed significant changes of expression (by a factor of ≥3-folds and *p*<0.05). All differentially expressed spots were identified by a MALDI-TOF/TOF mass spectrometer, and submitted to the NCBI nr database or the NCBI EST_ database. 163 differentially expressed proteins were successfully identified (Figure 2), among which 44 were identified as MRJPs, and 119 were clustered to 101 non-redundant proteins. The up-regulated and down-regulated proteins from the 3-day old *Ac* and *Am* larvae fed with RJC or RJM, and with CSBV infection were listed (Table 3).

**Table 3:** Identification of differentially expressed proteins.

No.		Protein Name	Gene	Accession No.	Protein MW (kDa)/PI					Protein Score C.I. %	GO	EC No.				Fold change
**AC-RJM-up**																
22	SF2 CG6987-PA		*SF2*	gi|66548276	28.4/9.88					100	F:nucleotide binding	**-**				↑∞
98	vasa intronic gene CG4170-PA, isoform A		*Vig*	gi|66565092	46.9/8.85					99.69	P:RNA interference	**-**				3.22
104	glyceraldehyde-3-phosphate dehydrogenase 2 isoform 1		*Gapdh*	gi|48142692	35.8/8.11					100	F:NAD/NADP binding; P:glucose metabolic process	**EC:1.2.1.12**				3.21
**AC-RJM-down**																
15	Gamma-actin		*Act5C*	gi|178045	25.8/5.65					100	P:adherens junction organization; F:protein kinase binding;	**-**				↓∞
21	ubiquitin conjugating enzyme E2		*ben*	gi|156551253	17.3/5.71					100	P:response to anesthetic; F:ubiquitin-protein ligase activity;	**EC:6.3.2.19**				↓∞
77	abnormal CHEmotaxis family member (che-3) isoform 1		*LOC411639*	gi|110757336	451.7/6.14					97.72	P:regulation of transcription, DNA-dependent; F:microtubule motor activity;	**EC:3.6.1.3**				↓∞
**AM-RJC-up**																
54	pORF2		*pORF2*	gi|16508047	149.5/9.7				99.97		P:RNA-dependent DNA replication; F:RNA-directed DNA polymerase activity;		**-**			↑∞
56	yellow-e2 CG17044-PA		*yellow-e2*	gi|110776421	39.6/8.29				99.39		P:defense response to fungus/G+/G-; caste determination, influence by environmental factors;		**-**			↑∞
63	Hypothetical protein Bd0095		*Bd0095*	gi|42521740	41.4/6.17				95		-		**-**			↑∞
64	mitochondrial malate dehydrogenase precursor isoform 1		*LOC408950*	gi|66513092	35.8/9.33				100		P:tricarboxylic acid cycle; F:L-malate dehydrogenase activity;		**EC:1.1.1.37**			↑∞
85	Ornithine aminotransferase precursor CG8782-PA		*Oat*	gi|66524972	47.3/8.5				99.99		P:metabolic process; F:ornithine-oxo-acid transaminase activity;		**EC:2.6.1.13**			↑∞
158	40S ribosomal protein S12		*RpS*12	gi|66548338	15.3/5.48				100		P:translation; F:structural constituent of ribosome;		**-**			5.14
**AM-RJC-down**																
67	Heat shock protein cognate 5 CG8542-PA		*Hsc70-5*	gi|66501507	75.4/6.38			99.9			P:protein folding; F:2-alkenal reductase [NAD(P)] activity;		**EC: 1.3**			↓∞
68	thioredoxin peroxidase 1 CG1633-PA, isoform 1		*Tpx-1*	gi|66548188	21.7/5.65			100			P:oxidation-reduction process; F:glutathione/thioredoxin peroxidase activity;		**EC: 1.1**			↓∞
69	Elongation factor 2 (EF-2) isoform 1		*Ef*2*b*	gi|66508439	94.5/6.11			100			P: translational elongation; F:translation elongation factor activity;		**-**			↓∞
70	Fumarylacetoacetase (Fumarylacetoacetate- hydrolase)(Beta-diketonase)		*Faa*	gi|66522657	41.6/6.09			100			P:aromatic amino acid family metabolic process; F:fumarylacetoacetase activity;		**EC: 3.7**			↓∞
71	Putative invasion protein		*invA*	gi|16266003	7.4/9.64			97.68			P:nucleobase-containing compound metabolic process; F:hydrolase activity, acting on acid anhydrides, in phosphorus-containing anhydrides		**EC: 3.6**			↓∞
86	T-complex Chaperonin 5 CG8439-PA, isoform A		*Cct*5	gi|66522349	59.4/5.7			97.50					**-**			↓∞
100	hypothetical protein LOC726980		*CG13321*	gi|110755727	14.4/5.52			99.99			F:transferase activity		**-**			3.21
101	40S ribosomal protein S12		*Rps12*	gi|66548338	15.3/5.48			100			P:translation; F:structural constituent of ribosome;		**-**			3.19
102	60 kDa heat shock protein, mitochondrial-like		*Hsp60*	gi|66547450	60.4/5.64			100			P:response to stress; F:solute:hydrogen antiporter activity;		**-**			6.64
103	Glyceraldehyde 3 phosphate dehydrogenase 1		*Gapdh*1	gi|66517066	31.6/7.6			100			P:glucose metabolic process; F:glyceraldehyde-3-phosphate dehydrogenase (NAD+) (phosphorylating) activity;		**EC: 1.2**			3.27
136	hexamerin 110		*Hex110*	gi|155369750	112.1/6.43			99.98			-		**-**			3.21
**AC-RJC-CSBV-up**																
8	Vacuolar h		*Vha55*	gi|66531434	55.1/5.41				99.57		P:ATP metabolic process; F:hydrogen-exporting ATPase activity, phosphorylative mechanism;		**EC: 3.6**			↑∞
9	HSP70		*hsp70*	gi|92430370	70.8/5.42				99.97		P:response to stress; F:ATP binding;		**-**			↑∞
10	40S ribosomal protein S3a (C3 protein)		*RpS3A*	gi|66547340	30.0/9.67				100		F:structural constituent of ribosome; P:translation		**-**			↑∞
72	MTA1-like CG2244-PB, isoform B		*MTA*1*-like*	gi|110756803	94.4/9.68				95.86		F:zinc ion binding; P:regulation of transcription, DNA-dependent		**-**			↑∞
73	peroxidase-like isoform 2		*pxd*	gi|110757934	87.6/7.11				99.053		P:response to oxidative stress; F:peroxidase activity;		**EC: 1.1**			↑∞
74	Argonaute 1 CG6671-PB, isoform B		*AGO*1	gi|110777044	103.6/9.35				98.599		P:targeting of mRNA for destruction involved in RNA interference; F:miRNA binding;		**-**			↑∞
93	phosphatidylethanolamine-binding protein homolog F40A3.3-like isoform 1		*LOC408516*	gi|66524882	20.6/8.84				99.793		-		**-**			3.55
133	60S ribosomal protein L9		*RpL9*	gi|66565444	21507.7/9.88				99.962				**-**			3.17
132	10 kDa heat shock protein, mitochondrial-like		*LOC552531*	gi|66547447	11.4/8.01				100		P:response to stress; F:ATP binding;		**-**			5.25
**AC-RJC-CSBV-down**																
7	Hypothetical protein V12B01_09746		*V12B01_09746*	gi|84388955		20.5/5.61				99.616	-	**-**				↓∞
89	tyrosyl-trna cytoplasmic		*Aats-tyr*	gi|110762892		73.7/8.91				99.548	P:tyrosyl-tRNA aminoacylation; F:tyrosine-tRNA ligase activity;	**EC: 6.1**				3.64
91,128	fructose-bisphosphate aldolase		*FBA*	gi|110748949		39.6/7.57				99.959	P:glycolysis; F:fructose-bisphosphate aldolase activity;	**EC: 4.1**				3.79 3.50
92	aldose reductase-like isoform 1		*AKR*-1	gi|66525576		36.1/6.26				99.997	P:oxidation-reduction process; F:oxidoreductase activity	**-**				3.97
120	ribosomal protein s9		*RpS*9	gi|48101950		22.5/10.74				97.835	P:translation; F:rRNA binding	**-**				4.47
122	peroxiredoxin 1		*Jafrac*1	gi|66548188		21.7/5.65				95.152	P:hydrogen peroxide catabolic process; F: thioredoxin/glutathione peroxidase activity;	**EC: 1.1**				8.45
124	phosphoglycerate kinase		*Pgk*	gi|110763826		44.9/8.15				100	P:phosphorylation; F:phosphoglycerate kinase activity;	**EC: 2.7**				9.74
125	60 kDa heat shock protein, mitochondrial-like		*Hsp60*	gi|66547450		60.4/5.64				100	P:response to stress; F:solute:hydrogen antiporter activity;	**-**				3.45
126	t-box protein h15		*mid*	gi|110757297		9.7/9.36				99.914	F:sequence-specific DNA binding transcription factor activity; P:regulation of transcription, DNA-dependent;	**-**				14.10
127	prohibitin protein wph		*wph*	gi|48097857		29.9/6.54				100	Required for larval metabolism or for the progression of the larva into a pupa	**-**				3.72
129	pyruvate kinase		*LOC552007*	gi|66548684		55.8/6.92				100	P:phosphorylation; F:potassium ion binding;	**EC: 2.7**				3.93
131	similar to CG31531-PA, isoform A isoform 1		*LOC409705*	gi|66500352		113.1/6.62				98.889	P:imaginal disc-derived wing morphogenesis; F:zinc ion binding	**-**				3.09
**AC-RJM-CSBV-up**																
13	eukaryotic translation initiation factor 5a		*eIF-5A*	gi|110767655		17.6/5.19				97.017	F:translation initiation factor activity; P:translational initiation;	**-**				↑∞
14	Triosephosphate isomerase1		*Tpi*	gi|148224276		26.9/7.77				99.999	P:gluconeogenesis; F:triose-phosphate isomerase activity	**EC: 5.3**				↑∞
87	neurochondrin homolog		*CG2330RA*	gi|66509434		84.2/5.89				99.177	P:metabolic process; F:phosphoglycolate phosphatase activity	**EC: 3.1**				3.24
135	actin, indirect flight muscle-like		*Act88F*	gi|110775533		37.3/5.37				99.948	P:phagocytosis, engulfment; F:ATP binding;	**-**				3.04
**AC-RJM-CSBV-down**																
75	grip and coiled-coil domain-containing protein 1		*CG10703*	gi|110750733		58.3/8.69				95.142.	P:cellular process	**-**				↓∞
76	60 kDa heat shock protein, mitochondrial precursor (Hsp60)		*HSP60*	gi|66547450		60.4/5.64				100	P:response to stress; F:transporter activity;	**-**				↓∞
134	yellow-e2 CG17044-PA		*CG15040*	gi|110776421		39.6/8.29				99.773	P:killing of cells of other organism;	**-**				3.36
**AM-RJC-CSBV-up**																
31		lambda-crystallin homolog	*Cryl1*	gi|66521282		34.4/6.3		100			F:3-hydroxyacyl-CoA dehydrogenase activity; P:oxidation-reduction process		**EC:1.1.1.35**			↑∞
32		GL19229	*Dper\GL19229*	gi|194106483		17.3/6.05		99.505			-		**-**			↑∞
34		Catalase	*Cat*	gi|25990773		57.9/8.39		100			P:hydrogen peroxide catabolic process; F:protein kinase activity;		**EC:1.11.1.6**			↑∞
82		metallo-beta-lactamase domain-containing protein 1-like	*CG9117*	gi|110775625		16.1/5.86		95.029			F:hydrolase activity		**-**			↑∞
83		transposase	*LOC725919*	gi|110766128		22.6/9.6		98.985			P:transposition, DNA-mediated; F:transposase activity;		**-**			↑∞
84		Translation elongation factor eEF-1 alpha chain	*Eif*	gi|58585198		50.5/9.16		99.948			F:translation elongation factor activity; P:translational elongation;		**-**			↑∞
108		40S ribosomal protein S20	*RpS20*	gi|110766823		13.7/9.95		100			P:translation; F:structural constituent of ribosome;		**-**			6.08
109		serine protease easter	*Sp*7	gi|110757145		41.0/7.83		98.199			P:proteolysis; F:serine-type endopeptidase activity;		**EC:3.4.21.0**			5.08
110		atp synthase subunit mitochondrial	*LOC551766*	gi|110762902		55.1/5.25		100			P:ATP synthesis coupled proton transport; F:ATP binding;		**EC:3.6.3.6**			3.80
111		CuZn superoxide dismutase	*Sod*	gi|33089104		15.6/6.21		100			P:superoxide metabolic process; F:superoxide dismutase activity;		**EC:1.15.1.1; EC:1.11.1.7**			4.13
112		Nucleoside diphosphate kinase (NDK) (NDP kinase) (Abnormal wing disks protein) (Killer of prune protein)	*Awd*	gi|66520497		17.6/6.75		100			P:nucleoside diphosphate phosphorylation; F:nucleoside diphosphate kinase activity;		**EC:2.7.4.6**			4.15
113		fructose-bisphosphate aldolase-like	*FBA*	gi|110748949		39.6/7.57		100			P:glycolysis; F:fructose-bisphosphate aldolase activity		**EC:4.1.2.13**			3.49
114		ornithine aminotransferase, mitochondrial	*Oat*	gi|66524972		47.3/8.5		100			P:metabolic process; F:ornithine-oxo-acid transaminase activity;		**EC:2.6.1.13**			4.46
33, 115,116,118		glyceraldehyde-3-phosphate dehydrogenase	*LOC410122*	gi|66517066		31.6/7.6		100			F:NAD/NADP binding; P:glucose metabolic process;		**EC:1.2.1.12**			↑∞ 4.29 3.16 3.89
117		larval cuticle protein a3a-like	*CPR31A*	gi|110764443		34.3/5.54		100			F:structural constituent of cuticle		**-**			3.25
119,137,138		glyceraldehyde-3-phosphate dehydrogenase 2 isoform 1	*Gapdh*	gi|48142692		35.8/8.11		100			F:NAD/NADP binding; P:glucose metabolic process;		**EC:1.2.1.12**			8.54 3.08 3.14
**AM-RJC-CSBV-down**																
24, 25, 26	60 kDa heat shock protein, mitochondrial precursor		*Hsp60*	gi|66547450		60.4/5.64	100				P:response to stress; F:transporter activity		**-**			↓∞ ↓∞ ↓∞
27	60 kDa heat shock protein, mitochondrial		*Hsp60*	gi|170045840		60.4/5.39	100				P: protein metabolic process; response to obiotic stimulus; F: nucleotide/protein binding; catalytic activity;		**EC: 4.2**			↓∞
30	GI16304		*Hsp60*	gi|193906980		60.7/5.65	99.812				P: protein metabolic process; response to obiotic stimulus; F: nucleotide/protein binding; catalytic activity;		**EC: 4.2**			↓∞
78	Spectrin beta chain		*Beta-Spec*	gi|110759783		268.6/5.37	97.139				F:phosphoprotein phosphatase activity;		**EC: 3.1**			↓∞
80	Stretchin-Mlck CG18255-PA, isoform A		*Strn-Mlck*	gi|110757372		38.1/5.45	99.212				-		**-**			↓∞
81	yellow-h CG1629-PA		*Yellow-h*	gi|110761428		33.7/5.71	100				-		**-**			↓∞
105	fatty acid binding protein		*FABP*	gi|27531027		15.5/5.46	100				F:lipid binding; P:transport;		**-**			8.52
107	protein NPC2 homolog		*NPC2*	gi|110756609		16.1/7.55	100				-		**-**			3.22
**AM-RJM-CSBV-up**																
50	c6 transcription		*SS1G_11931*	gi|156039589		50.2/8.99	97.085				F:sequence-specific DNA binding RNA polymerase II transcription factor activity; P:regulation of transcription from RNA polymerase II promoter;		-	↑∞		
51,161	protein disulfide-isomerase		*Pdi*	gi|110768510		24.3/4.74	100				F:electron carrier activity; P:cell redox homeostasis;		**EC: 5.3; EC: 2.4**		↑∞ 18.95	
52	aldehyde dehydrogenase mitochondrial-like		*Aldh*	gi|66526635		45.4/5.25	100				F:retinal dehydrogenase activity; P:oxidation-reduction process		**EC: 1.2**		↑∞	
53	hydroxysteroid dehydrogenase-like protein 2-like		*CG5590*	gi|66530010		45.2/8.69	100				P:oxidation-reduction process; F:glucose 1-dehydrogenase [NAD(P)] activity;		**EC: 1.1**		↑∞	
153	heat shock protein cognate 3		*Hsc70-3*	gi|229892214		72.8/5.29	100				F:metal ion binding; P:porphyrin-containing compound biosynthetic process;		**EC: 4.2**		4.15	
154	phosphoglycerate kinase isoform 1		*PGK*	gi|110763826		44.9/8.15	100				P:glycolysis; phosphorylation; F:phosphoglycerate kinase activity		**EC: 2.7**		6.13	
155	a-kinase anchor protein 9		*Cp309*	gi|110758909		81.4/8.39	98.602				P:microtubule cytoskeleton organization; F:protein binding;		**-**		7.52	
156	heat shock protein cognate 4		*Hsc70-4*	gi|229892210		71.0/5.43	100				F:ATP binding; P:response to stress;		**-**		4.99	
157	glyceraldehyde-3-phosphate dehydrogenase 2 isoform 1		*Gapdh*	gi|48142692		35.9/8.11	100				F:NAD/NADP binding; glyceraldehyde-3-phosphate dehydrogenase (NAD+) (phosphorylating) activity; P:oxidation-reduction process;		**EC: 1.2**		3.68	
161	26S proteasome non-ATPase regulatory subunit 13		*Rpn9*	gi|66547365		43.6/6.07	95.252				-		**-**		18.95	
163	myosin heavy muscle isoform 1		*Mhc*	gi|110759191		224.7/5.78	99.86				P:ecdysone-mediated induction of salivary gland cell autophagic cell death; F:2-alkenal reductase [NAD(P)] activity;		**EC: 1.3**		3.14	
**AM-RJM-CSBV-down**																
35	30S ribosomal protein S3		*RpS*3	gi|73661978		24.0/9.8	99.657				P:translation; F:RNA binding;		**-**		↓∞	
37, 142,143	thioredoxin peroxidase 1 CG1633-PA		*Jafrac*1	gi|66548188		21.8/5.65	100				P:response to stress; F:antioxidant activity;		**EC: 1.1**		↓∞, 3.36, 4.50	
38	aldo-keto reductase		*CG6084*	gi|66525576		36.1/6.26	100				P:metabolic process; F:catalytic activity		**-**		↓∞	
36,39,42 141, 144145148	60 kDa heat shock protein, mitochondrial precursor		*Hsp60*	gi|66547450		60.4/5.64	100				P:response to stress; F:transporter activity;		**-**		↓∞ ↓∞ ↓∞ 7.55 3.95 29.95 3.24	
40	tyrosyl-trna cytoplasmic		*Aats-tyr*	gi|110762892		73.7/8.91	100				P:translation; F:catalytic activity; RNA binding;		**EC: 6.1**		↓∞	
43, 46	heat shock protein 60		*Hsp60*	gi|156541962		60.5/5.5	100				P:response to stress; F:transporter activity;		**-**		↓∞ ↓∞	
47	fructose-bisphosphate aldolase		*FBA*	gi|110748949		39.6/7.57	100				P:carbohydrate metabolic process; F:catalytic activity		**EC: 4.1**		↓∞	
49	heat shock cognate 70		*Hsp70*	gi|66537940		71.7/5.43	100				P:response to stress; F:nucleotide binding;		**-**		↓∞	
139	arylphorin subunit alpha		*Lsp2*	gi|149939401		79.5/6.72	100				-		**-**		4.00	
140	atp synthase subunit mitochondrial		*Vha68*	gi|110762902		55.1/5.25	100				P:generation of precursor metabolites and energy; F:hydrolase activity;		**EC: 3.6**		3.07	
147	similar to Pyruvate kinase CG7070-PB, isoform B		*LOC552007*	gi|66548684		55.8/6.92	100								3.99	
149	hexamerin 110		*Hex110*	gi|155369750		112.1/6.43	100				-		**-**		4.27	
150	protein NPC2 homolog		*NPC2*	gi|110756609		16.1/7.55	100				-		**-**		6.33	
151	grpe protein mitochondrial-like		*Roe*1	gi|66525522		26.4/7.82	100				P:protein metabolic process; F:protein binding;		**-**		3.66	
152	n-acetylneuraminate lyase-like		*LOC725646*	gi|110755974		34.0/8.3	100				P:metabolic process; F:catalytic activity		**-**		4.19	

Spot numbers and folds change correspond to the number of the protein spots in Figure 2. The theoretical molecular weight (Mr) and isoelectric point (pI) of the identified proteins were retrieved from the protein database of NCBInr limited to *Apis mellifera*. The Mascot score was searched against the *A. mellifera* database. Protein name is given when proteins were identified by MALDI-TOF/TOF-MS. The taxonomy is *A. mellifera*. Accession number is the unique number given to mark the entry of a protein in the NCBInr database. GO molecular function and biology process result in the Blast2GO analysis, P, Biological process; F, Molecular function.

**Figure 3:**
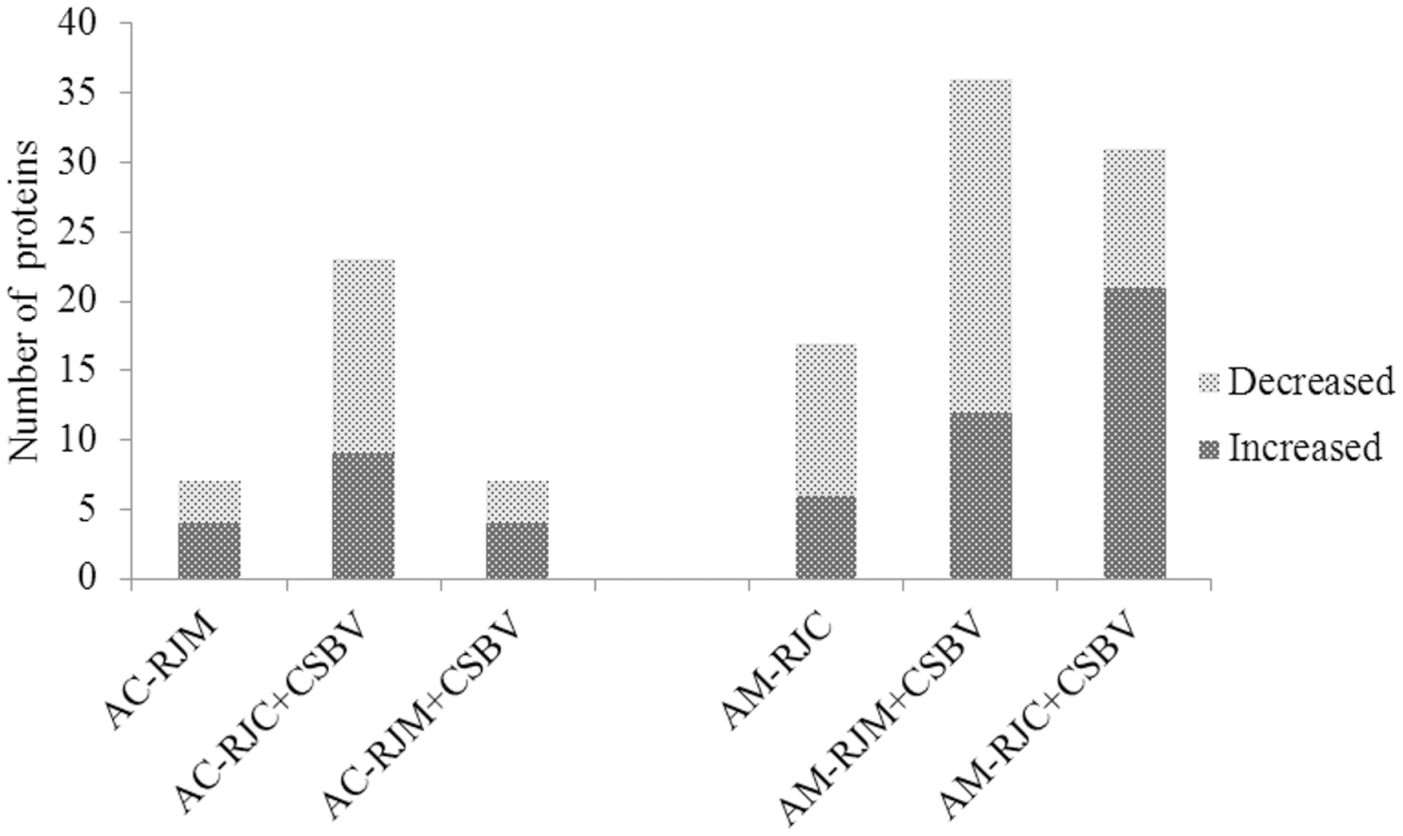
Numbers of up-regulated and down-regulated proteins from the bee larvae fed with RJs and challenged by CSBV. On the basis of triplicate replications analyses, only the proteins that changed ≥3.0-fold in relative ratios (p<0.05) were considered. RJC: royal jelly from *A. cerana*; RJM: royal jelly from *A. mellifera*; AC-RJM: 3-day old *Ac* larvae fed with RJM. AC-RJC+CSBV: 3-day old *Ac* larvae fed with RJC and infected with CSBV. AC-RJM+CSBV: 3-day old *Ac* larvae fed with RJM and infected with CSBV. AM-RJC: 3-day old *Am* larvae fed with RJC. AM-RJC+CSBV: 3-day old *Am* larvae fed with RJC and infected with CSBV. AM-RJM+CSBV: 3-day old *Am* larvae fed with RJM and infected with CSBV.

In *Ac* larvae, 6 differential expression proteins (6 spots) were identified from heterospecific RJ breeding only, 21 differential expression proteins (22 spots) from CSBV challenge only and 7 differential expression proteins (7 spots) from heterospecific RJ breeding plus CSBV challenge. In *Am* larvae, 17 differential expression proteins (17 spots) were identified from heterospecific RJ breeding only, 26 differential expression proteins (36 spots) from CSBV challenge only and 24 differential expression proteins (31 spots) from heterospecific RJ breeding plus CSBV challenge (Figure 3).

Some proteins were not identified either because their abundance was too low to produce enough spectra or because the database search scores can not yield unambiguous results (>95%).

### GO (Gene Ontology) Functional Term Enrichment

**Figure 4:**
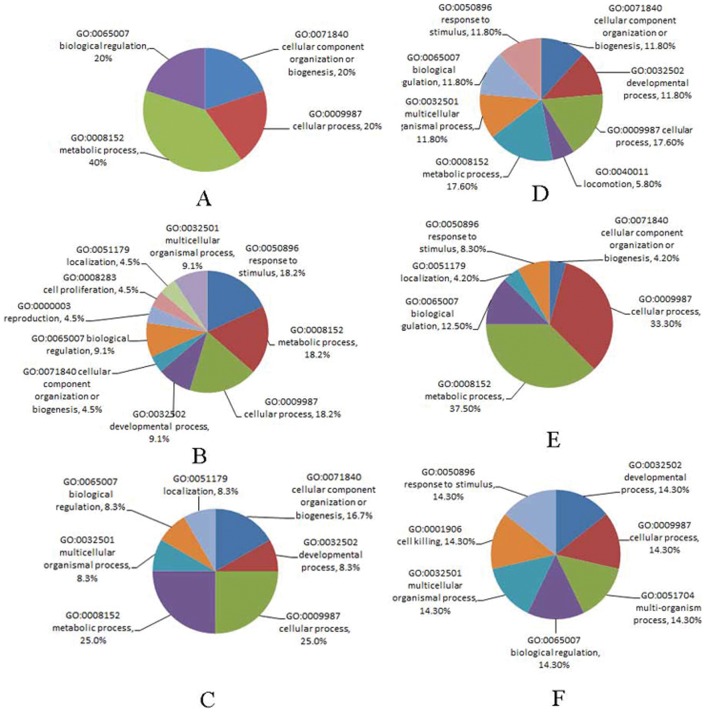
Based on the GO annotations, the proteins from *A. cerana* larvae fed with RJM or RJC, and by CSBV challenge, were grouped by the broad biological processes (BP). A, B, C: up-regulated proteins; D, E, F: down-regulated proteins. RJC: royal jelly from *A. cerana*; RJM: royal jelly from *A. mellifera*; A, D: 3-day *Ac* larvae fed with RJM; B, E: 3-day *Ac* larvae fed with RJC, and infected with CSBV; C, F: 3-day *Ac* larvae fed with RJM, and infected with CSBV.

**Figure 5:**
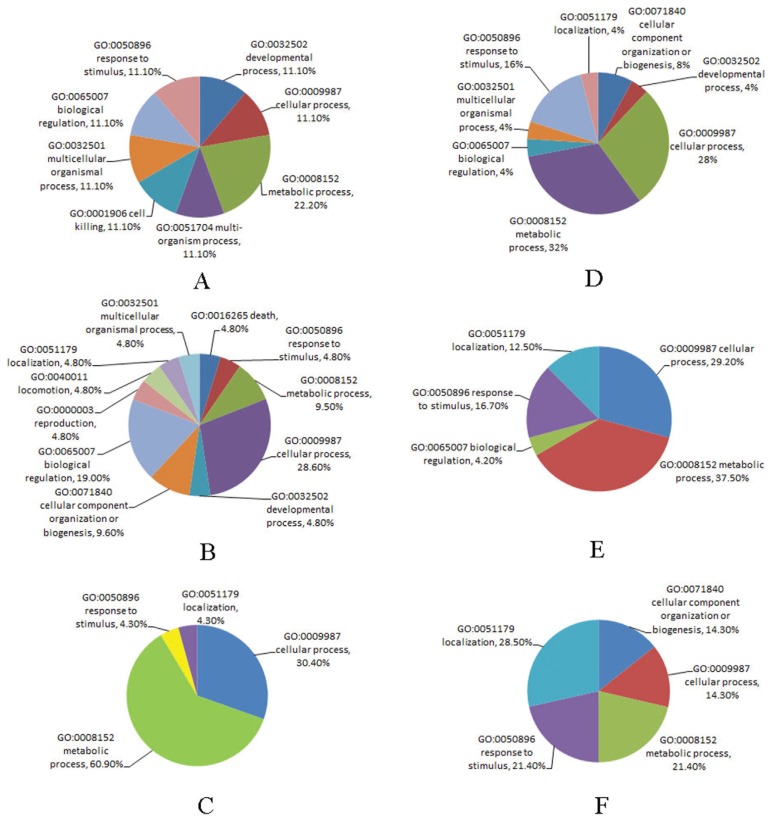
Based on the GO annotations, the proteins from *A. mellifera* larvae fed with RJM or RJC, and by CSBV challenge, were grouped by the broad biological processes (BP). A, B, C: up-regulated proteins; D, E, F: down-regulated proteins. RJC: royal jelly from *A. cerana*; RJM: royal jelly from *A. mellifera*; A, D: 3-day *Am* larvae fed with RJC; B, E: 3-day *Am* larvae fed with RJM, and infected with CSBV; C, F: 3-day *Am* larvae fed with RJC, and infected with CSBV.

To understand the functions of differentially expressed proteins from the bee larvae, which resulted from heterospecific RJ breeding or CSBV infection, 101 identified proteins were analysed by gene ontology (GO) mapping respectively. At least one GO term could be assigned to 88 of 101 detected differentially expressed proteins (Table 3). Thirteen (12.8%) proteins were deemed of unknown function after blast searches against the nr databases and annotation augmentation. The GO terms assigned to individual proteins were shown in Table 3. Based on the GO annotations, proteins were grouped by their broad biological processes (BP) (Figure 4, 5).

Three major BPs of the up-regulated proteins from the *Ac* larvae fed with RJM were metabolic processes (40.0%), cellular processes (20%) and biological regulation (20%), whereas those of down-regulated proteins were mainly metabolic processes (17.6%), cellular processes (17.6%) and developmental process (11.8%). The major BPs of the increasing proteins from *Ac* fed with RJC, and then by CSBV challenge were metabolic process (18.2%), cellular processes (18.2%) and response to stimulus (18.2%), whereas the major BPs of the decreasing proteins were metabolic processes (37.5%), cellular processes (33.3%) and biological regulation (12.5%). The major BPs of the increasing proteins from *Ac* fed with RJM, and then by CSBV challenge were metabolic processes (25.0%), cellular processes (25.0%) and cellular component organization or biogenesis (16.7%), whereas the major BPs of the decreasing proteins were metabolic processes (25.0%), cellular processes (25.0%) and biological regulation (12.5%).

Three major BPs of the increasing proteins from the *Am* larvae fed with RJM were metabolic processes (22.2%), cellular processes (11.1%) and cell killing (11.1%), whereas those of decreasing proteins were metabolic processes (32.0%), cellular processes (28.0%) and response to stimulus (16%). The major BPs of the increasing proteins from *Am* fed with RJM, and then by CSBV challenge were cellular processes (28.6%), biological regulation (19.0%) and cellular component organization (9.6%), whereas the major BPs of the decreasing proteins were metabolic processes (37.5%), cellular processes (29.2%) and response to stimulus (16.7%). The major BPs of the increasing proteins from *Am* fed with RJC, and then by CSBV challenge were metabolic processes (60.9%), cellular processes (30.4%) and response to stimulus (4.3%), whereas the major BPs of the decreasing proteins were localization (28.5%), response to stimulus (21.4%) and metabolic processes (21.4%).

### Network Analysis of Differentially Expressed Proteins

**Figure 6:**
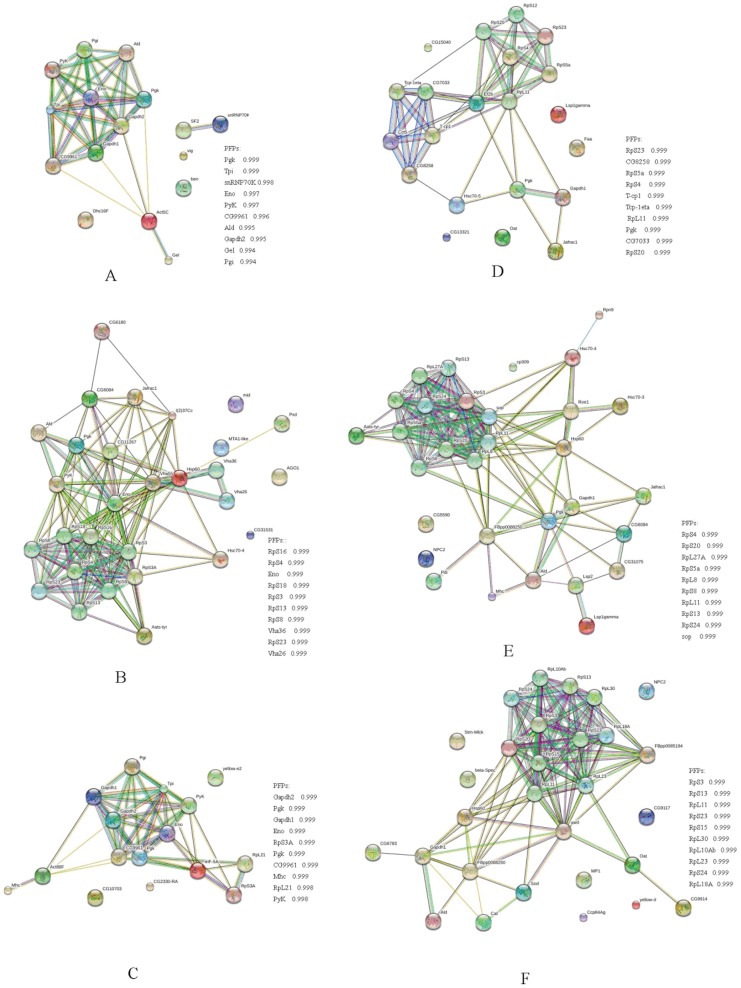
Protein-protein interaction networks of identified differentially expressed proteins from nutritional crossbreeding and CSBV challenge were constructed by Search Tool for the Retrieval of Interacting Genes (STRING). Lines represent the existence of the different types of evidence used in predicting the associations. A red line indicates the presence of fusion evidence; a green line, neighborhood evidence; a blue line, co-occurrence evidence; a purple line, experimental evidence; a yellow line, text mining evidence; a light blue line, database evidence; a black line, coexpression evidence. PFPs: Predicted Functional Partners. A: 3-day *Ac* larvae fed with RJM. B: 3-day *Ac* larvae fed with RJC, and infected with CSBV. C: 3-day *Ac* larvae fed with RJM, and infected with CSBV. D: 3-day *Am* larvae fed with RJC. E: 3-day *Am* larvae fed with RJM, and infected with CSBV; F: 3-day *Am* larvae fed with RJC, and infected with CSBV.

Proteins function jointly in a living cell through networks by forming protein- protein interactions (PPI), modifications and regulation of expression relationships. Of all the identified proteins in this study, 107 proteins with the established functions based on *Drosophila melanogaster* were selected and their PPI mapping was constructed using STRING. The result showed that all the treats almost divided into two distinct PPI clusters with several others operating without connectivity. The first cluster primarily consisted of cytoskeletal proteins, while the second cluster mainly consisted of proteins involved in metabolism (Figure 6).

### Verification of Differentially Expressed Proteins

**Figure 7:**
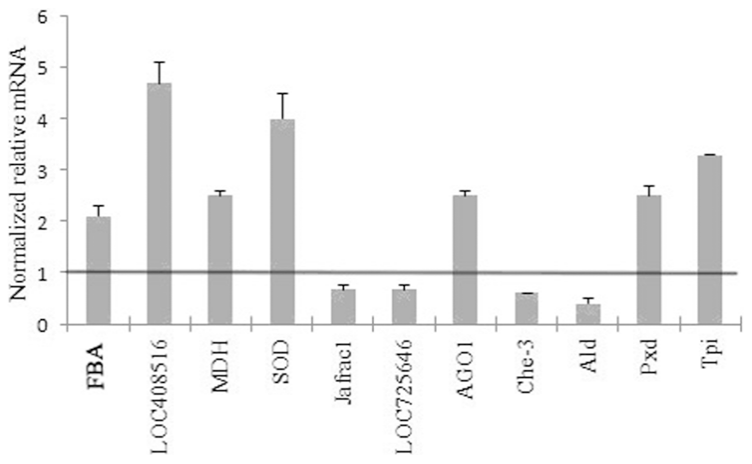
Verification of 11 differentially expressed proteins at mRNA level by qRT-PCR analysis. The normalized relative mRNA levels (>1) of the genes of the corresponding differentially expressed proteins indicate up-regulation and those (<1) indicate down-regulation. Error bar is standard deviation. The names of the proteins are referred in Table 2.

To test the tendency of protein expression between its encoding gene at the transcript level, 11 proteins (FBA, LOC408516, MDH, SOD, Jafrac1, LOC725646, AGO1, Che-3, Ald, Pxd and Tpi) were randomly selected for qRT-PCR analysis of their encoding genes. The trend of mRNA expression of the selected genes showed consistent patterns with their protein expression (Figure 7).

### Western Blot Analysis

**Figure 8:**
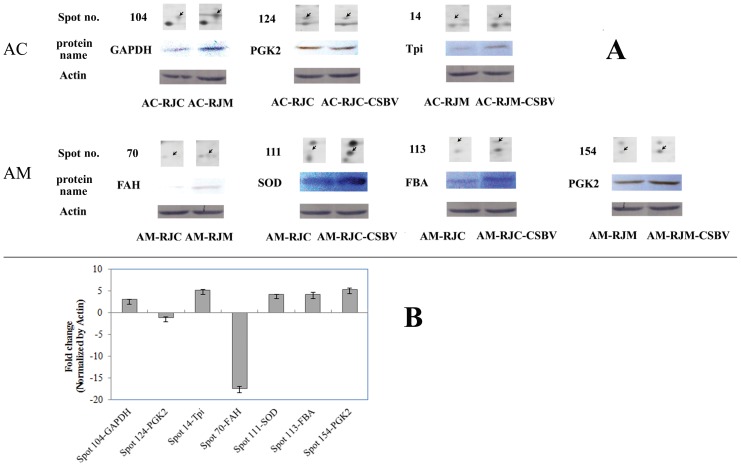
Western blot Analysis of differentially expressed proteins. Six proteins were selected. Proteins were separated on a 12% SDS-PAGE gel and immunoblotted with anti-GAPDH, anti-PKG2, anti-Tpi, anti-FAH, anti-SOD, and anti-FBA. β-actin was used as an internal control. (A) The Western blot images of GAPDH, PKG2, TPI, FAH, SOD, FBA and Actin. The protein spots from Figure 2. (B) Quantification of target protein expression.The relative fold change of GAPDH, PKG2, Tpi, FAH, SOD, FBA (normalized by Actin) was visulized by using Quantity One Software. The expression of GAPDH, SOD, FBA and Tpi were up-regulatedd. On the other hand, FAH and PGK were down-regulated. Error bar is standard deviation.

Western blot analysis was conducted to verify the expression of the proteins which are closely related to energy metabolism of heterospecific royal jelly and CSBV resistance, such as GAPDH, PGK, Tpi, FAH, SOD, and FBA. The results are consistent well with the proteomic data (Figure 8).

## Discussion

Heterospecific RJ breeding means during the growth or reproduction period of the colony or individual, changing the comb from *Ac* to *Am* or from *Am* to *Ac* or feeding RJ artificially. In recent years, heterospecific RJ breeding between *Ac* and *Am* has been concerned in the bee industry [39]. This technology was used between different species of western honeybees in 1957. *Am* fed with RJC showed some morphological characteristic of *Ac*, such as proboscis, forewings, abdomen and thorax, and body weight of new adults [23], [40], and even influenced the genotype of malate dehydrogenase II [41]. It was reported that *Am* could be resistant to varroa mites when fed with RJC [42]–[44]. Interestingly, the present results showed that the change of *Ac* larval food from RJC to RJM could enhance *Ac* resistance to CSBV, under the laboratory condition. The mortality of *Ac* larvae after CSBV infection was much lower when they were fed with RJM compared with those fed with RJC. However, the change of RJM to RJC did not lower the viral resistance of *Am* larvae. Whether RJM stimulates the viral resistance of *Ac* by inducing the antiviral protein expression or RJM molecules can directly inhibit the viral replication in bee larvae needs further study.

To determine whether the proteins induced by the food change are probably involved in the bee resistance to CSBV, 2-DE and MALDI-TOF-MS based proteomic strategies were used. Accordingly, 101 nonredundant proteins were identified as being differentially expressed among *Ac* and *Am* larvae by heterospecific RJ breeding and CSBV challenge.

### Differentially Expressed Proteins from Hererospecific RJ Breeding

The results showed that protein expression was influenced by the RJ. Within 6 differentially expressed proteins from 3-day old *Ac* larvae fed with RJM, 3 proteins CG6987-PA (SF2), vasa intronic gene (Vig), and GAPDH were up-regulated, and 3 proteins (Act5C, ben, Che-3) down-regulated. SF2 is a RNA recognition motif, a highly abundant domain in eukaryotes found in proteins involved in post-transcriptional gene expression processes [45]. Vig has been identified as a component of *Drosophila* RISC involved in RNA interference [46]. GAPDH is a key enzyme in the glycolytic pathway. So the up-regulated proteins are associated with gene expression process, RNA interference and glycolytic metabolism. Of the down-regulated proteins, Gamma-actin (Act5C) is an ubiquitous protein involved in the formation of filaments that are a major component of the cytoskeleton. Ubiquitin conjugating enzyme E2 (E2) is an ubiquitin conjugating enzyme [47], [48]. The ubiquitin proteasome system enables cells to selectively recognize and degrade damaged or potentially harmful proteins [49]. Abnormal CHEmotaxis family member (Che-3) is one of the AAA+ superfamily. In *Caenorhabditis elegans* Che-3 is specifically responsible for the retrograde transport of the anterograde motor, kinesin-II, and its cargo within sensory in chemosensory neurons [50]. Therefore, the down-regulated proteins are related with the cytoskeleton arrangement, protein metabolism and cellular processes.

Correspondingly, 17 differentially expressed proteins from 3-day old larvae of *Am* fed with RJC included 6 up-regulated (pORF2, yellow-e2, MDH, Oat, RpS12, one unknown protein), and 11 down-regulated proteins (Hsc70-5, Tpx-1, Ef2b, FAH, invA, Cct5, Rps12, Hsp60,Gapdh1, Hex110, one unknown protein). For the up-regulated proteins, pORF2 is a retrovirus reverse transcriptase with RNA-directed DNA polymerase activity [39]. Yellow-e2 responses to fungus/G^+^/G^−^, and functions in caste determination, and is influenced by environmental factors [51]. MDH is the key enzymes in the citric acid cycle, and possess L-malate dehydrogenase activity elevated by oxidative stress [52]. Ornithine aminotransferase precursor (Oat) possesses ornithine-oxo-acid transaminase activity in metabolic process [53]. For the down-regulated proteins, T-complex Chaperonin 5 (Cct5) is involved in productive folding of proteins [54]. The methylation of the *Hex110* gene in *A. mellifera* is regulated at the developmental stage and in a caste-dependent manner [55]. HSP60 and Hsc70-5 are heat shock proteins responding to stress. Generally, HSPs act as molecular chaperones facillitating protein maturation in cells and activating innate and acquired immune responses [6]. They are excellent immunomodulators against a wide variety of pathogens in protecting host [56]. Thioredoxin peroxidase 1 (Jafrac1) is in response to oxidative stress [57]. Elongation factor 2 (Ef2b) is a GTPase, involved in the translocation of the peptidyl-tRNA [58]. Fumarylacetoacetase hydrolysis (FAH) is responsible for the hydrolysis of fumarylacetoacetate to generate fumarate and acetoacetic acid [59]. Putative invasion protein (InvA) is a prominent inner-membrane component of the Salmonella type III secretion system (T3SS) apparatus, which is responsible for regulating virulence protein export in pathogenic bacteria [60].

From the above description, differentially expressed proteins from heterospecific RJ breeding were related to metabolism mainly in TCA and glycolytic pathway and response to microbe and oxidative stress. RJ protein difference between *Ac* and *Am* was reported [24]. The majority of the identified proteins were major royal jelly proteins (MRJPs) with MRJP1 being the most abundant. Peroxiredoxin 2540, glutathione S-transferase S1, and MRJP5 were detected only in the RJ of *A. mellifera ligustica*, and MRJP7 was found only in the RJ of *A. cerana cerana* [24]. Maybe different components of two royal jellies changed the metabolism pathways of honey bees, conferring to the resistant difference to viral infection.

### Differentially Expressed Proteins from *Ac* Larvae Fed with RJC and by CSBV Challenge

Comparing to the 3-day old *Ac* larvae fed with RJC and without CSBV infection, 21 altered proteins were detected from 3-day old *Ac* larvae fed with RJC and CSBV infection, in which 9 proteins being up-regulated (Vha55,Hsp70, RpS3A, MTA1-like, pxd, AGO1, phosphatidylethanolamine-binding protein, Hsp10, an unknown protein), and 12 proteins down-regulated (V12B01_09746, Aats-tyr, Ald, AKR-1, RpS9, Jafrac1, PGK, Hsp60, mid, wph, LOC552007, LOC409705).

The up-regulated proteins were mainly involved in the stress response and in the defense from viral infection. Such as, vacuolar h (Vha55) couples ATP hydrolysis to the build up of a H+ gradient. Metastasis associated gene 1 (MTA1) is part of the NURD (nucleosome remodeling and deacetylating) complex and plays a role in cellular transformation and metastasis [61]. Peroxidase-like (Pxd) is response to oxidative stress. Argonaute 1 (AGO1) is part of the RNA-induced silencing complex (RISC) in the RNA interference pathway which can defense insects from virus infection [62], [63].

The down-regulated proteins were mainly involved in the energy process and in the development regulation. For example, tyrosyl-trna cytoplasmic (Aats-tyr) are involved in amino acid synthesis, cell cycle control, RNA shear modification and transport of tRNA [64], [65]. Fructose-bisphosphate aldolase (FBA) and aldose reductase-like isoform 1 (AKR-1) are involved in glycolysis. PGK and pyruvate kinase are involved in energy metablism. PGK is the enzyme responsible for the first ATP generating step of glycolysis and the *in vivo* activation of l-nucleoside pro-drugs effective against retroviruses such as HIV and hepatitis [66]. Pyruvate kinase (PK) regulates the final rate-limiting step of glycolysis. In tumor cells, the glycolytic pyruvate kinase isoenzyme M2 (PKM2, M2-PK) determines whether glucose is converted to lactate for regeneration of energy or used for the synthesis of cell building blocks [67]. Cell proliferation only proceeds when metabolism is capable of providing a budget of metabolic intermediates that is adequate to ensure both energy regeneration and the synthesis of cell building blocks in sufficient amounts. In this study, the three important enzymes of glycolysis were significantly down regulated after *Ac* larvae fed with RJC and by CSBV challenge, indicating that the energy metabolism pathway may be important in antiviral activity of the bees. Prohibitin protein wph (Wph) is required for larval metabolism or for the progression of the larva into a pupa. LOC409705 is responsible for imaginal disc-derived wing morphogenesis. These proteins were significantly down-regulated, probably directly impacting the larval development.

As indicated in Figure 1, the *Ac* larvae fed with RJC were not resistant to CSBV infection. Although the up-and down-regulated protein expressions were detected from those bee larvae, it seemed that these proteins were not actively involved in effectively protecting the larvae from CSBV infection. Whether some *Ac* expressed proteins were active for helping viral infection needs further study.

### Differentially Expressed Proteins from *Ac* Larvae Fed with RJM and by CSBV Challenge

Comparing to the 3-day old *Ac* larvae fed with RJM but without CSBV infection, 4 highly expressed protein spots (eIF-5A, Tpi, CG2330RA, Act88F) and 3 down-regulated proteins (CG10703, HSP60, yellow-e2) were found from the *Ac* larvae fed with RJM and by CSBV infection.

Eukaryotic translation initiation factor 5a (eIF-5A) plays an important role in protein translation extending, and in stress tolerance [68]. Triosephosphate isomerase (Tpi) is a glycolytic enzyme [69]. Act88F (actin, indirect flight muscle-like) is involved in phagocytosis [51]. HSP 60 and yellow-e2 are both activated by environmental factors as discussed above. They were down-regulated may indicated that the influence of CSBV were weakened when the Ac larvae fed with RJM.

Interestingly, CSBV infection did not significantly increase the mortality of *Ac* larvae fed with RJM comparing to those fed with RJM and without viral challenge (Figure. 1). So RJM may protect *Ac* larvae from viral infection probably by promoting energy metabolism and activating phagocytosis. Another possibility is that the specific components from RJM, such as Peroxiredoxin 2540, glutathione S-transferase S1, and MRJP5, which are detected only in the RJ of *A. mellifera ligustica* [24], may inhibit the viral replication.

### Differentially Expressed Proteins from *Am* Larvae Fed with RJC and by CSBV Challenge

In comparison to 3-day old *Am* larvae fed with RJC and without CSBV infection, 24 proteins were detected from 3-day old *Am* larvae fed with RJC and CSBV infection, in which 17 proteins were up-regulated (Cryl1 (lambda-crystallin homolog), Dper\GL19229, Cat, CG9117 (metallo-beta-lactamase), LOC725919 (transposase), Eif, RpS20, serine protease easter (Sp7), LOC551766 (atp synthase subunit mitochondrial), SOD, Abnormal wing disks protein (Awd), FBA, ornithine aminotransferase (Oat), GADPH, larval cuticle protein a3a-like (CPR31A)) and 7 proteins down-regulated (Hsp60, Beta-Spec, Strn-Mlck, Yellow-h, FABP, NPC2, an unknown protein).

Most of the up-regulated proteins were involved in energy metabolism, such as Cryl1, CG9117, LOC725919, Sp7, LOC551766, FBA, Oat, LOC410122, and GAPDH. The regulation pattern of FBA and GADPH was opposite to that in the *Ac* larvae fed with RJC and challenged with CSBV, maybe due to different species of honey bees. But in response to viral invasion, two bee species showed the same way in regulation of antioxidative enzymes, such as SOD, CuZn superoxide dismutase and Catalase, which were biomarkers to evaluate the toxic effects of organisms [70]. The proteins (Awd and CPR31A) related to development were up-regulated, to some degree, but the cytoskeletal proteins (Beta-Spec and Strn-Mlck) were significantly down-regulated. These differentially expressed proteins might be involved in protecting *Am* larvae from CSBV infection, although these larvae were fed with RJC (Figure 1). *Am* larvae are not sensitive to CSBV infection [6]. The food change from RJM to RJC did not lower the resistant ability of *Am* to CSBV.

### Differentially Expressed Proteins from *Am* Larvae Fed with RJM and by CSBV Challenge

In response to CSBV challenge, 11 up-regulated proteins (SS1G_11931, Pdi, FBA, CG5590, Hsc70-3, PGK, Cp309, Hsc70-4, GAPDH, Rpn9, Mhc), and 15 down-regulated proteins (RpS3, Jafrac1, CG6084, Hsp60, Aats-tyr, ALD, Hsp70, Lsp2, Vha68, LOC552007, Hex110, NPC2, Roe1, LOC725646) were found from the larvae fed with RJM and with CSBV infection.

Among the up-regulated proteins, those involved in energy metabolism were the majority, including PDI, FBA, ALD, PGK and GAPDH. This was in according with the expression pattern of *Ac* larvae fed with RJM. 26S proteasome (Rpn9) plays a fundamental role in eukaryotic homeostasis [71], [72]. The ubiquitin-proteasome system mediated viral protein degradation constitutes a host defense process against some RNA viral infections [73]. In the present study Rpn9 and HSPs were both up-regulated, indicating the possible involvement of these proteins in antiviral activity. Correspondingly, chaperonins or store proteins were down-regulated. This may guarantee the normal growth of *Am* larvae. As a source of amino acids for tissue reconstruction during pupal development, hexamerins are first synthesized by a larval fat body and released into the hemolymph where they accumulate to extraordinarily high concentrations to build adult structures [74].

## Conclusions

Although *Ac* is frequently damaged by CSBV, *Am* is usually resistant to this virus. The present study showed that the change of *Ac* larval food from RJC to RJM could enhance the bee resistance to CSBV, at least at early larval stage. Proteomic technology was employed to unravel the molecular events of the bees under heterospecific RJ breeding and CSBV challenge. There were 101 proteins with altered expressions after heterospecific RJ breeding and viral infection in two bee species. The RJM may protect *Ac* larvae from CSBV infection, probably by activating the genes in energy metabolism pathways, antioxidation and ubiquitin-proteasome system. The results, for the first time, comprehensively descript the molecular events of the viral infection of *Ac* and *Am*, and are potentially useful for establishing CSBV resistant populations of *Ac* for apiculture.
